# Supplementary figures and images for: GnRH or estradiol benzoate combination with CIDR improves in-vivo embryo production in bovines (Bos indicus and Bos taurus) under subtropics
Source: PeerJ. 2021 Sep 16;9:e12077. doi: 10.7717/peerj.12077 (PMC8450005; doi:10.7717/peerj.12077)

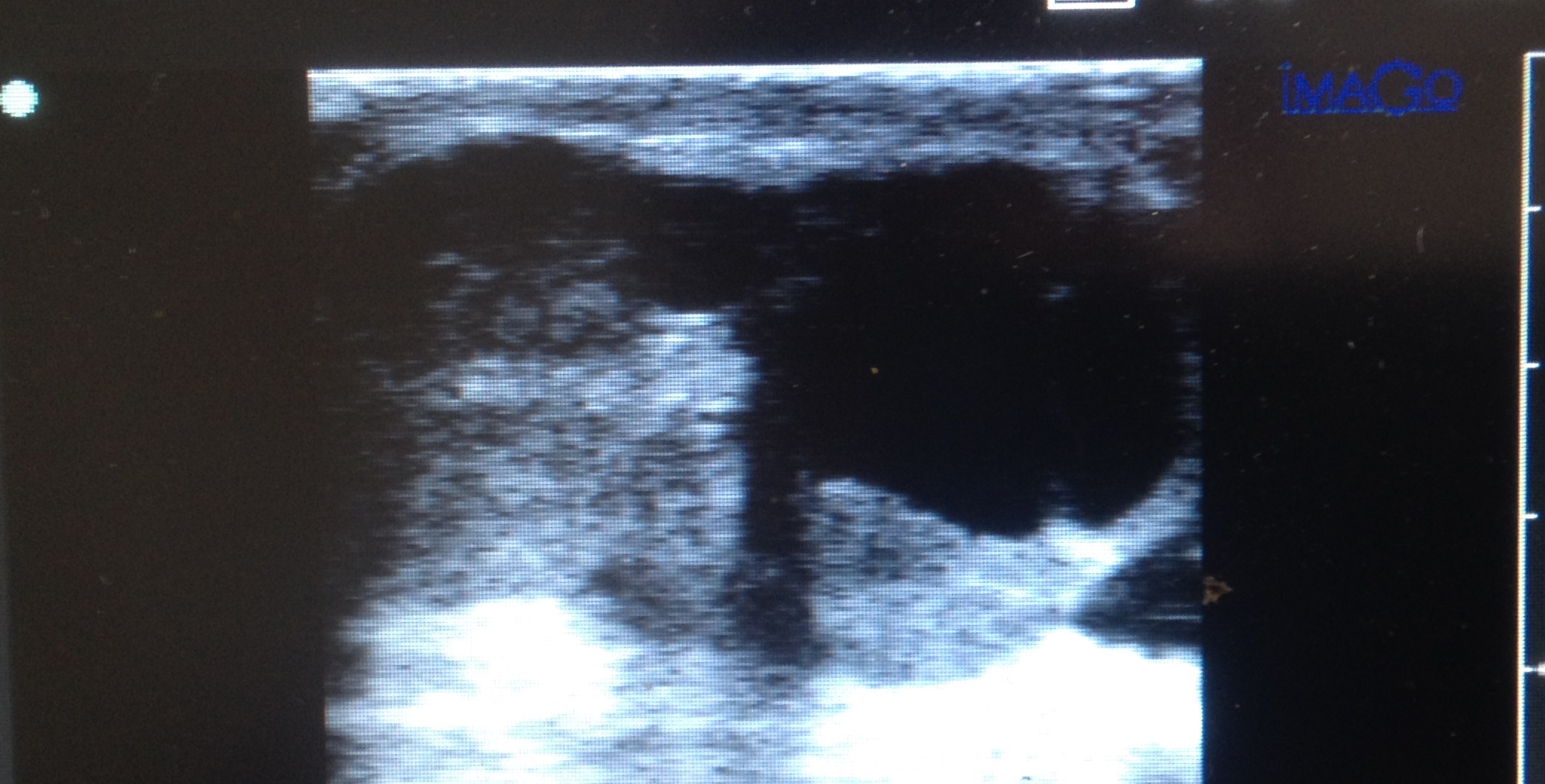

Supplement: Supplemental Information 4 [file peerj-09-12077-s004.jpg]

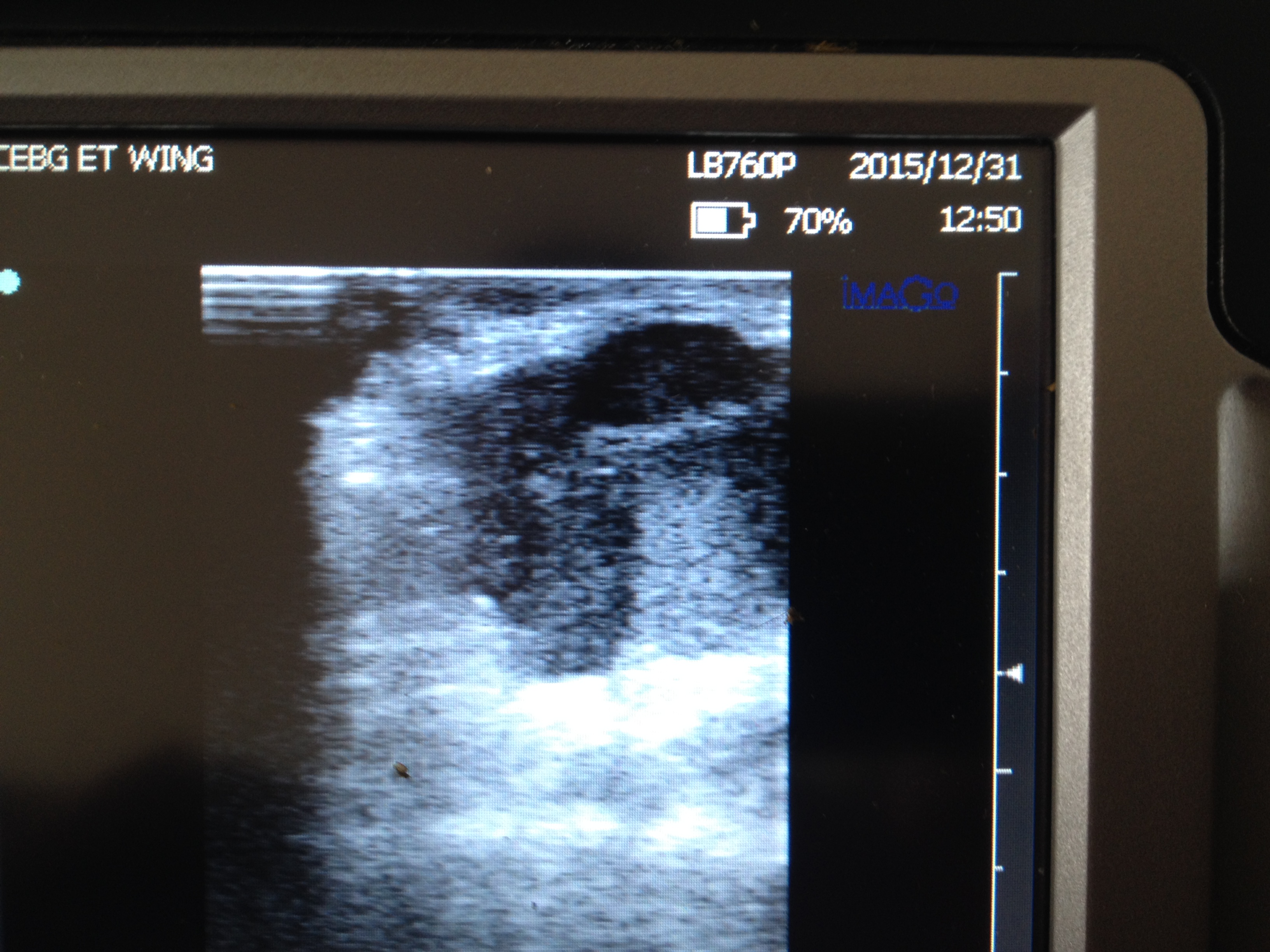

Supplement: Supplemental Information 5 [file peerj-09-12077-s005.jpg]

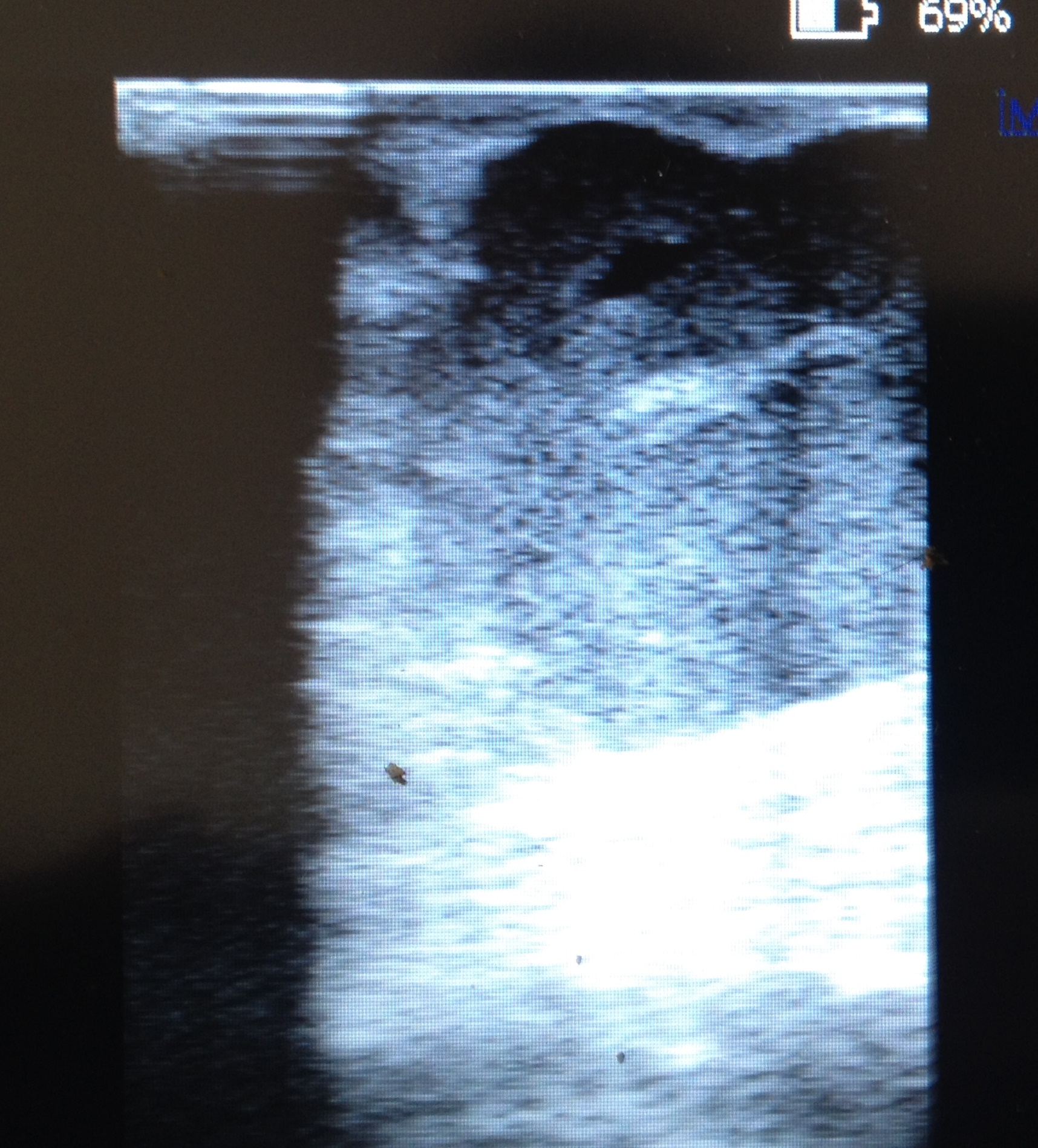

Supplement: Supplemental Information 6 [file peerj-09-12077-s006.jpg]

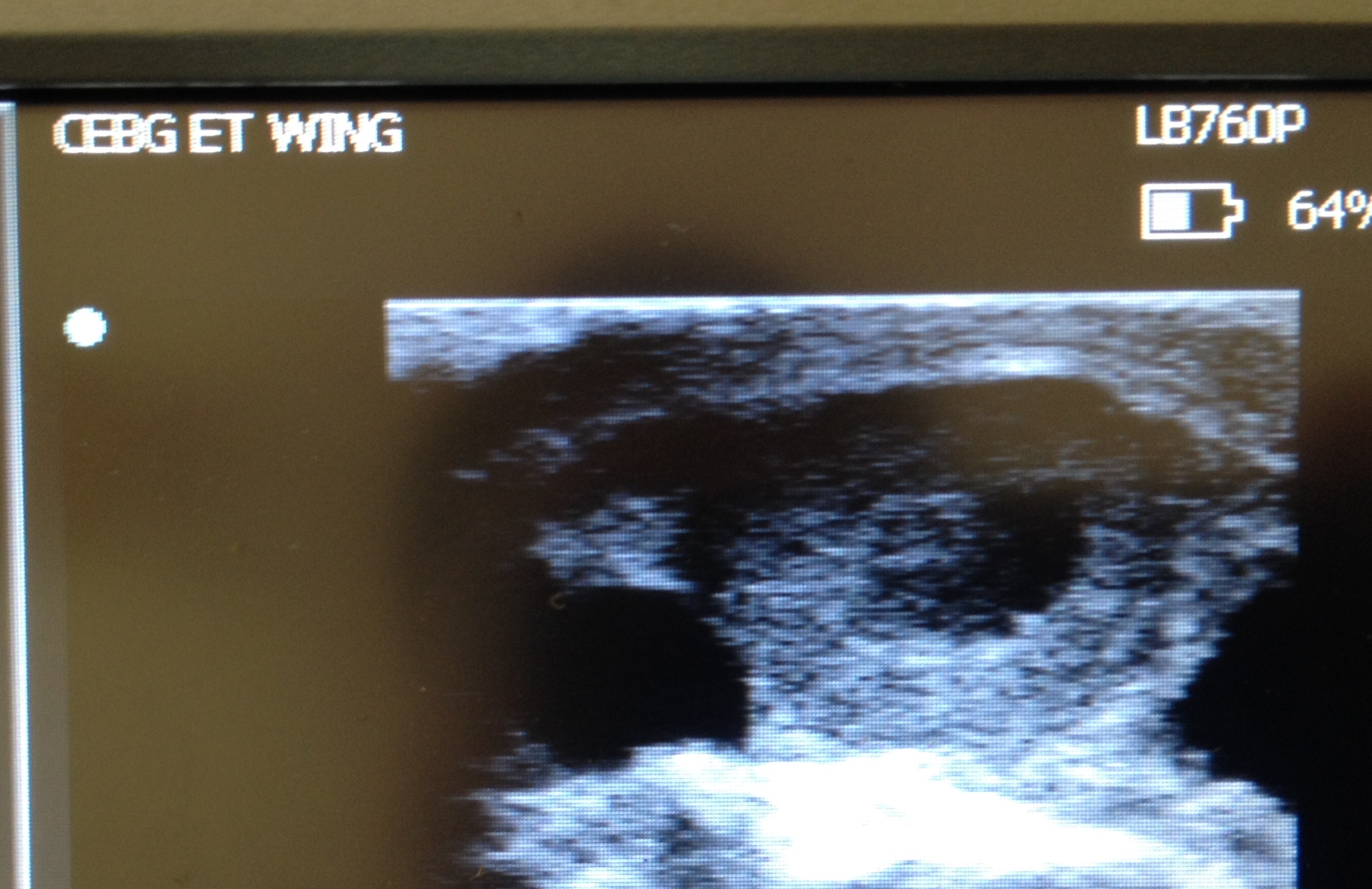

Supplement: Supplemental Information 7 [file peerj-09-12077-s007.jpg]

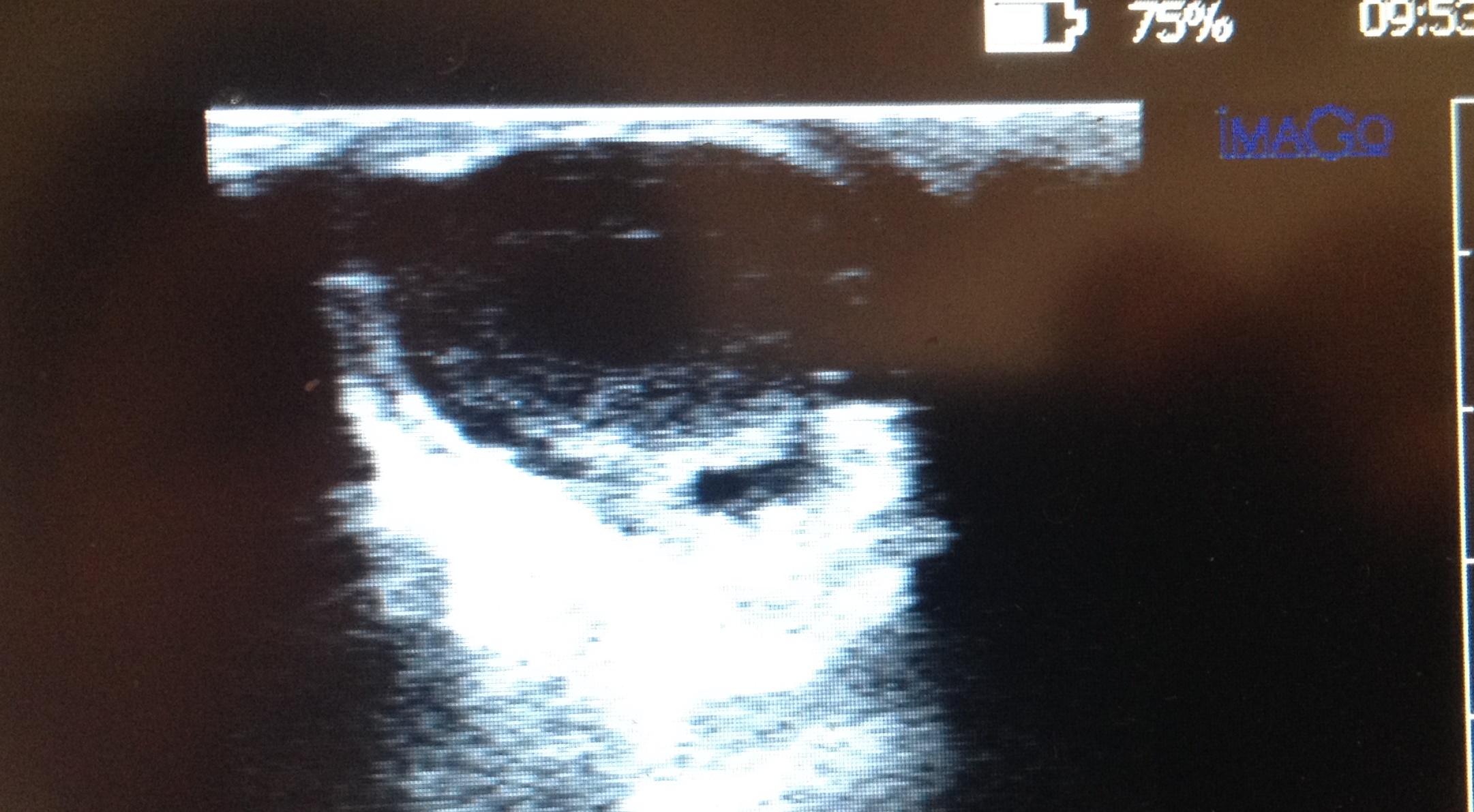

Supplement: Supplemental Information 8 [file peerj-09-12077-s008.jpg]

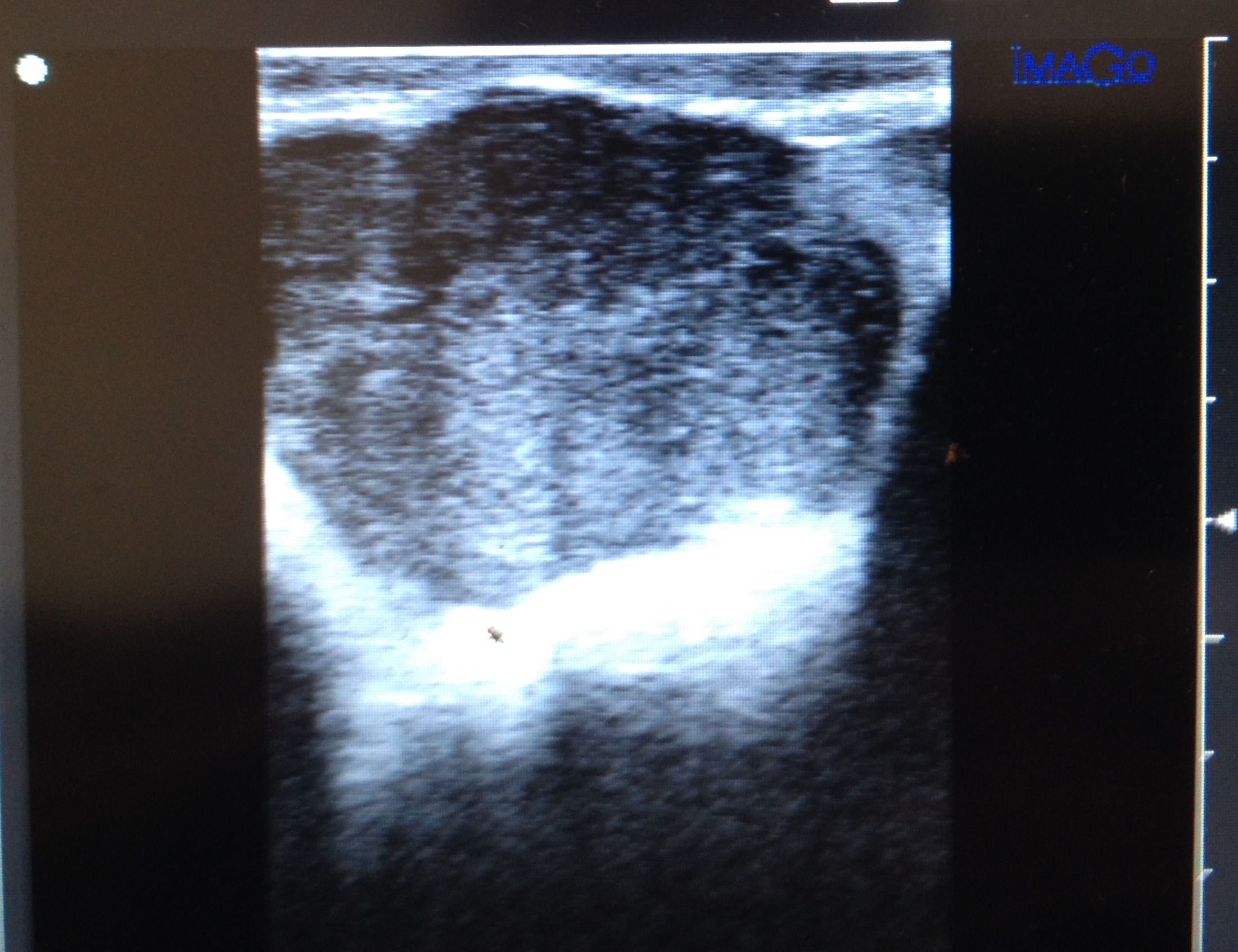

Supplement: Supplemental Information 9 [file peerj-09-12077-s009.jpg]

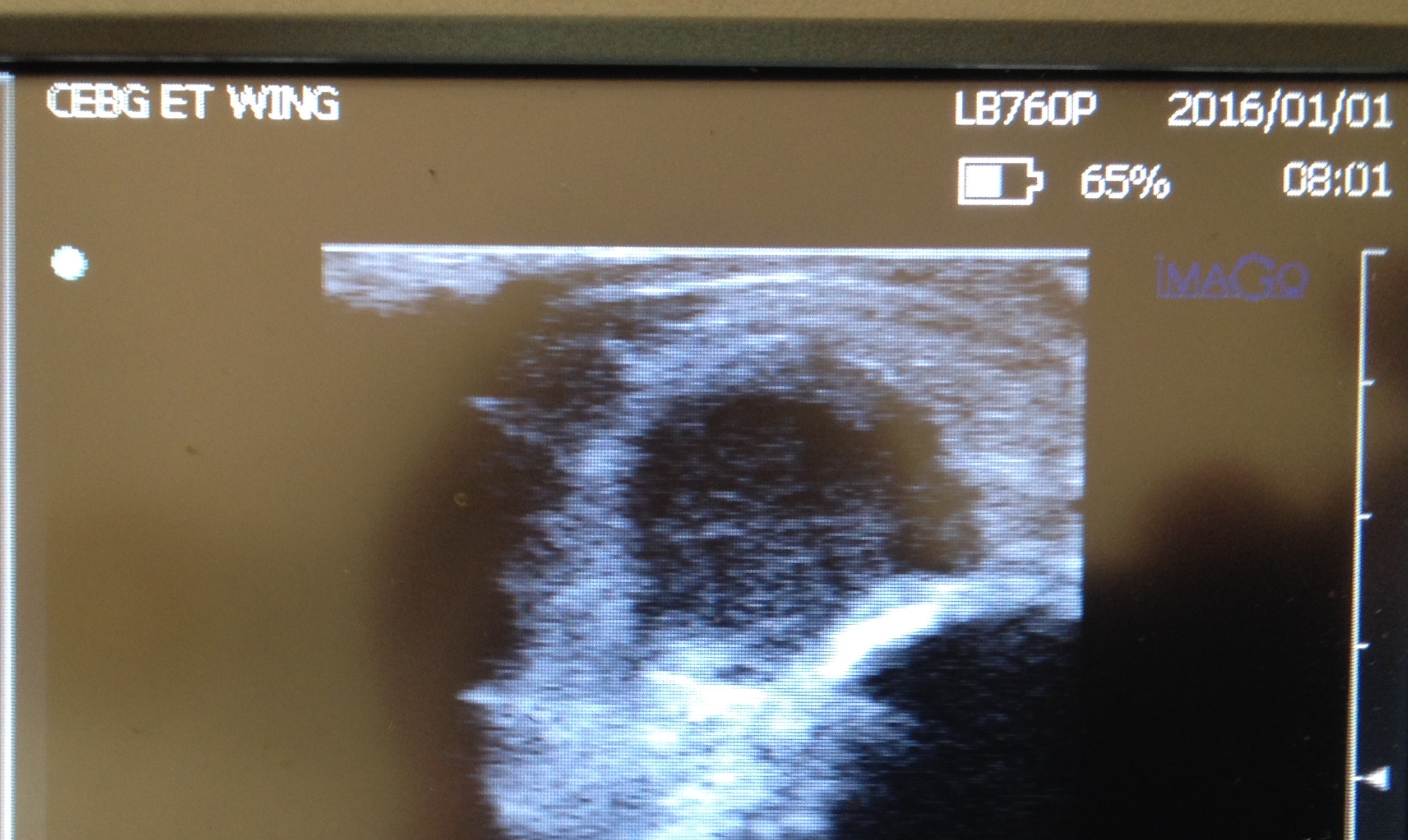

Supplement: Supplemental Information 10 [file peerj-09-12077-s010.jpg]

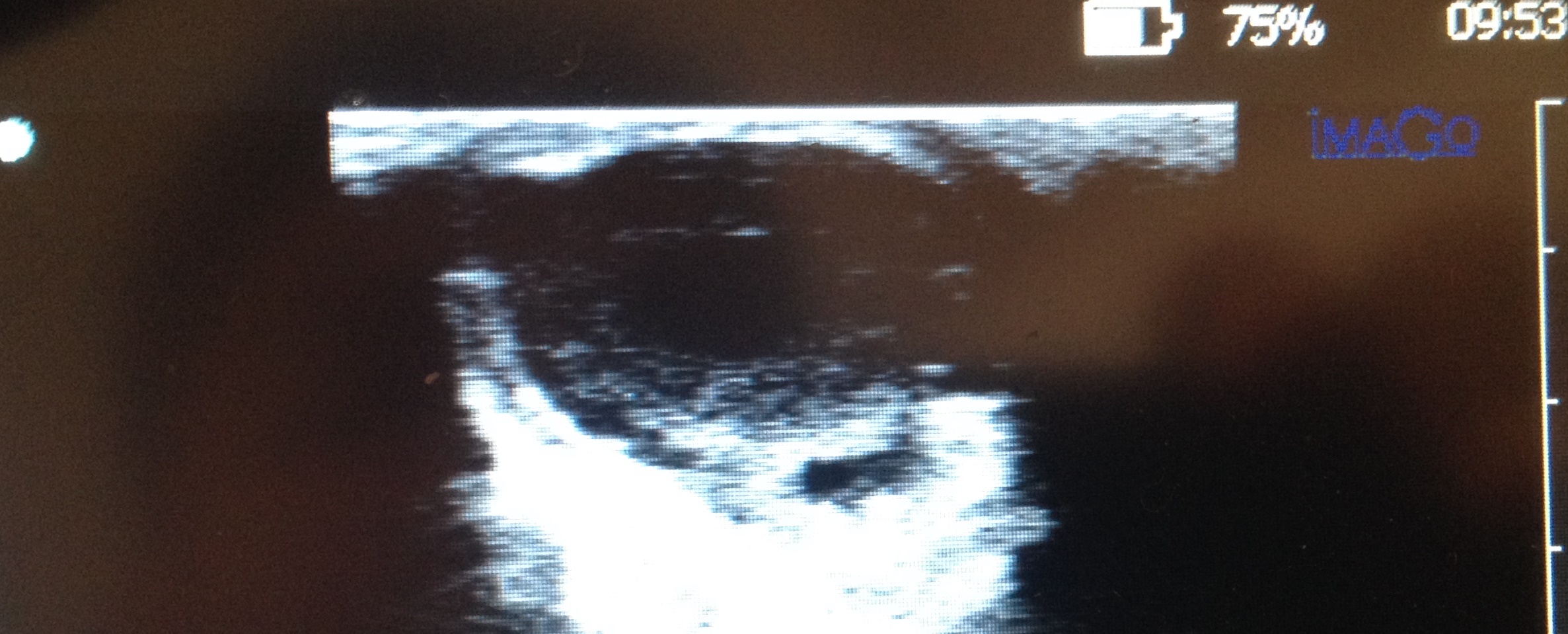

Supplement: Supplemental Information 11 [file peerj-09-12077-s011.jpg]

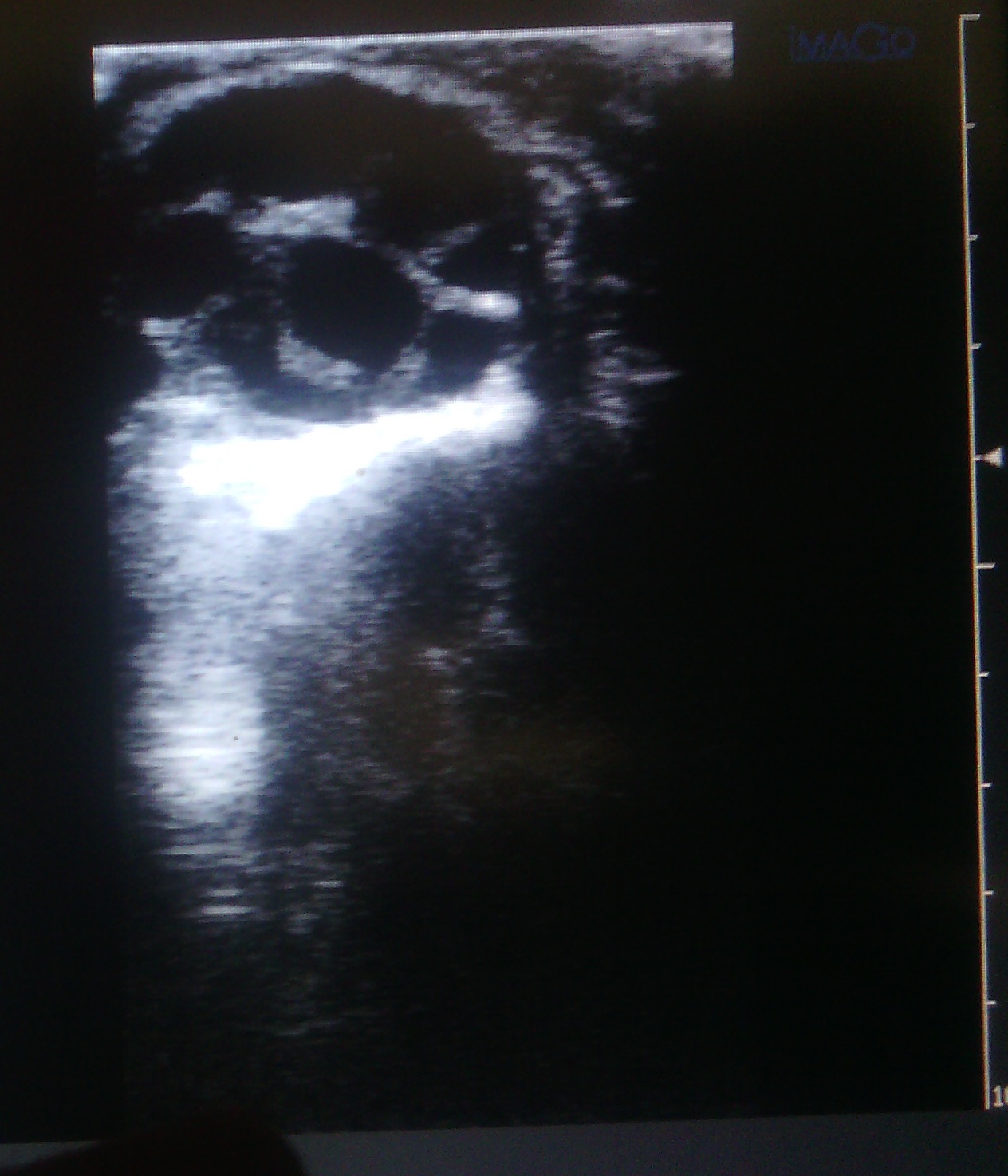

Supplement: Supplemental Information 12 [file peerj-09-12077-s012.jpg]

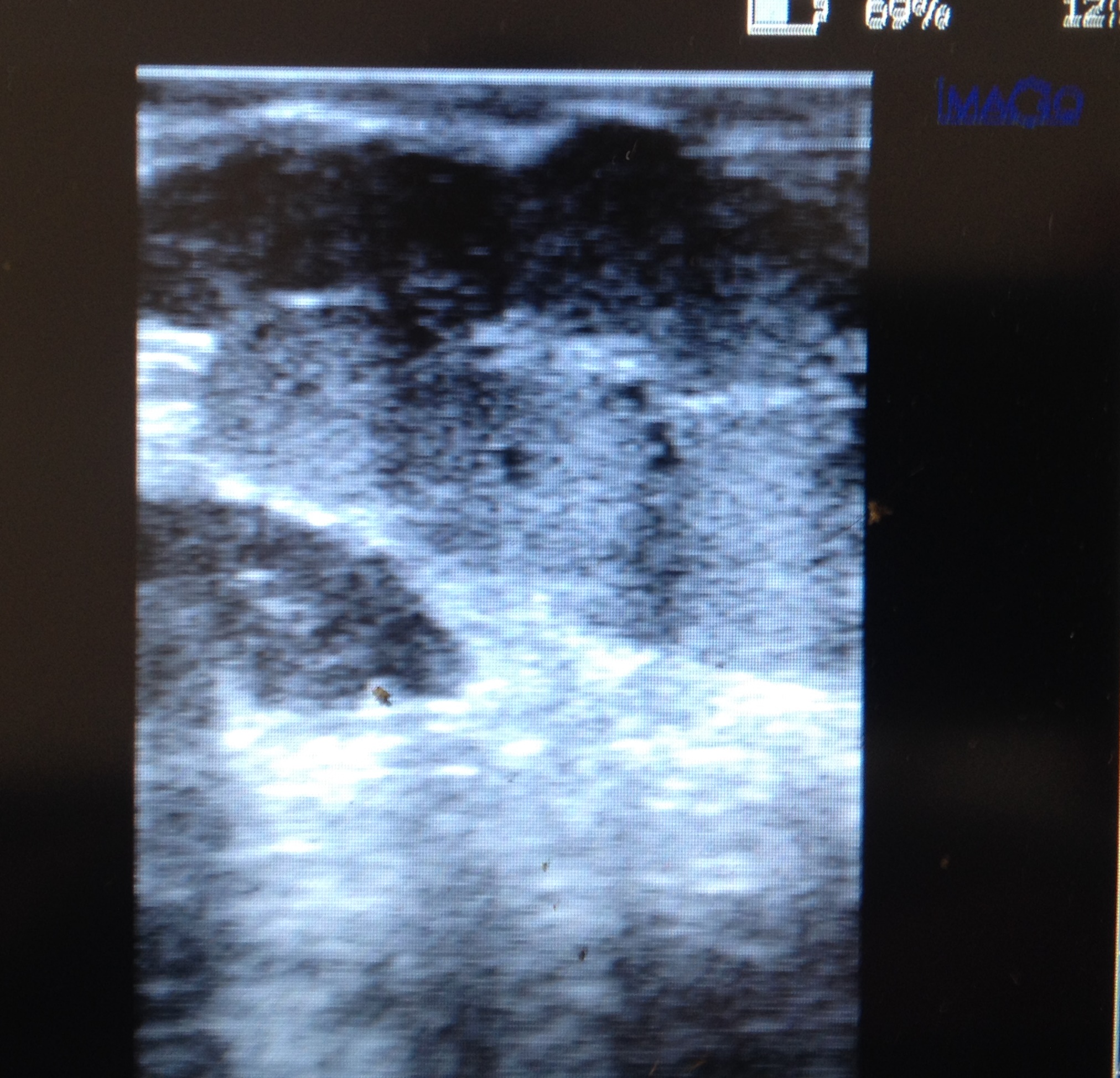

Supplement: Supplemental Information 13 [file peerj-09-12077-s013.jpg]

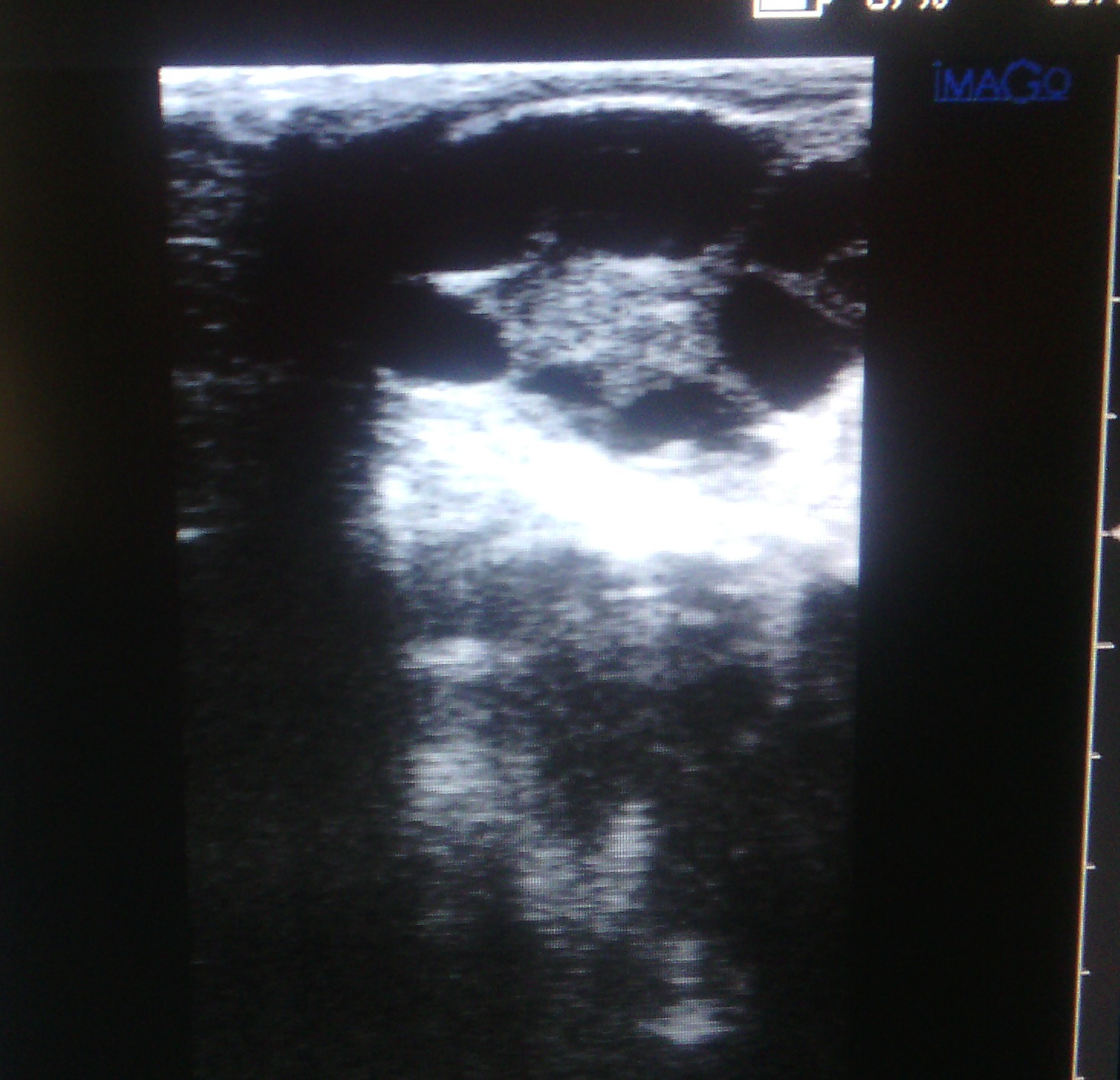

Supplement: Supplemental Information 14 [file peerj-09-12077-s014.jpg]

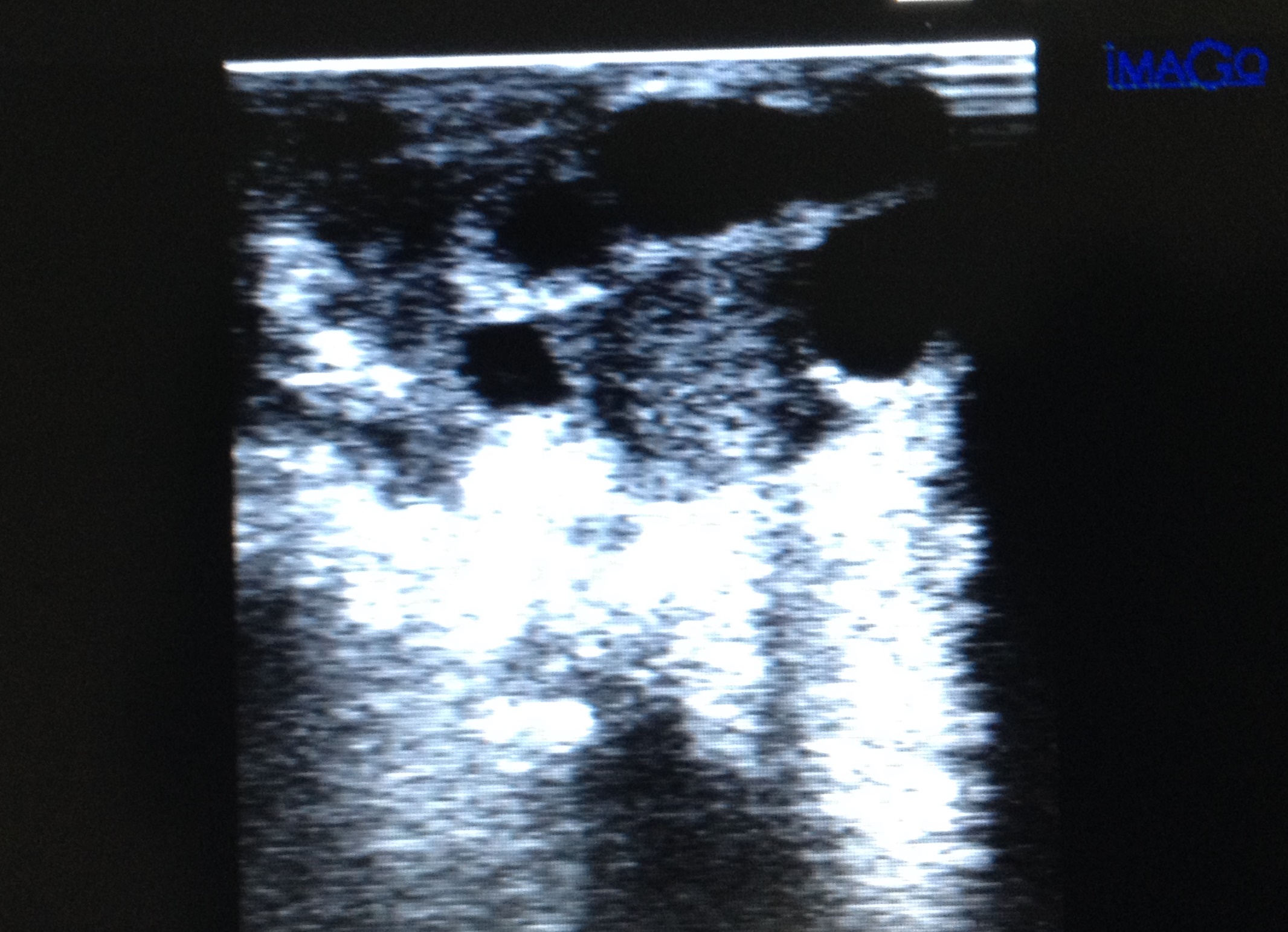

Supplement: Supplemental Information 15 [file peerj-09-12077-s015.jpg]

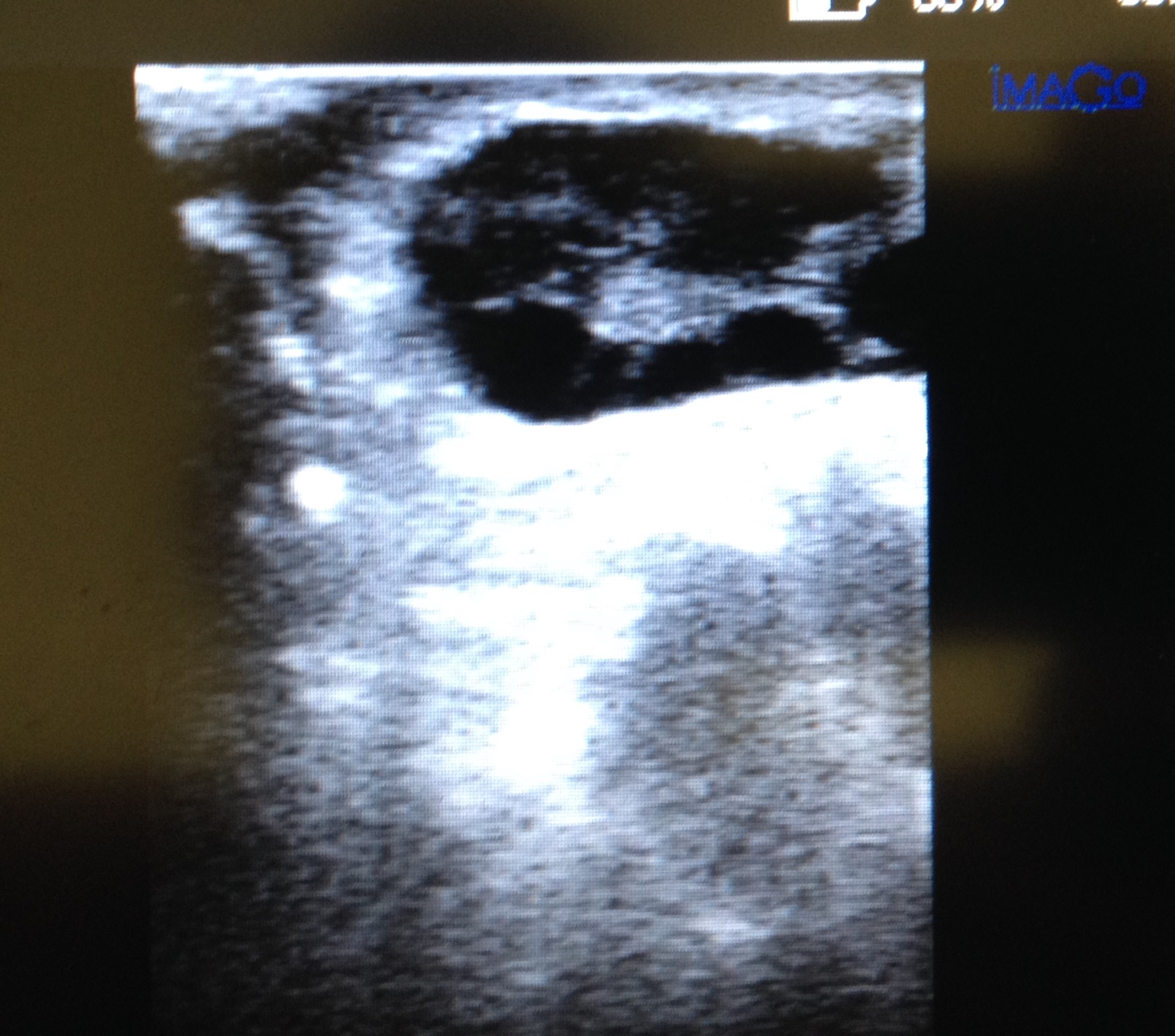

Supplement: Supplemental Information 16 [file peerj-09-12077-s016.jpg]

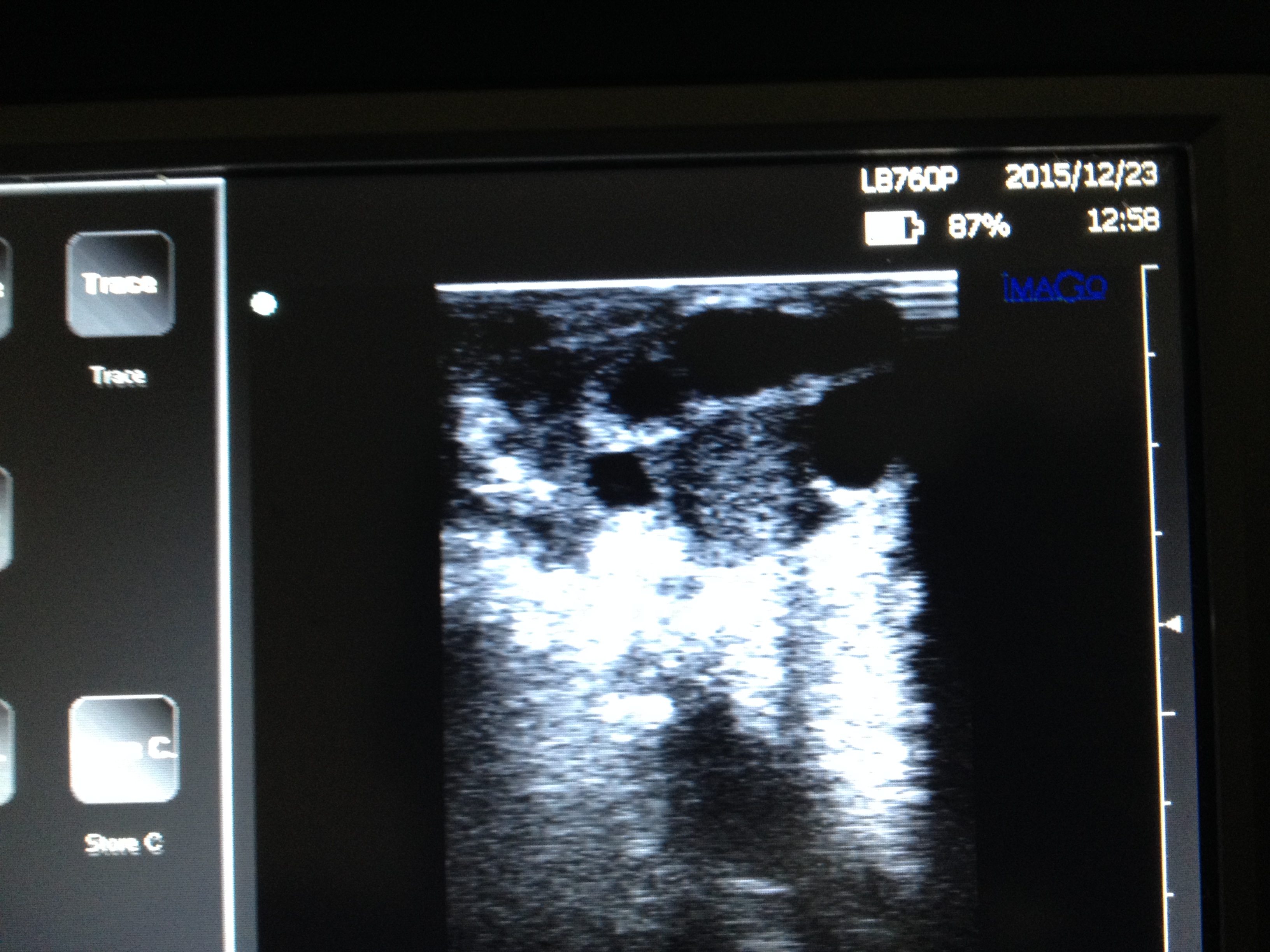

Supplement: Supplemental Information 17 [file peerj-09-12077-s017.jpg]
