# Supplementary material for: GnRH or estradiol benzoate combination with CIDR improves in-vivo embryo production in bovines (Bos indicus and Bos taurus) under subtropics
Source: PeerJ. 2021 Sep 16;9:e12077. doi: 10.7717/peerj.12077 (PMC8450005; doi:10.7717/peerj.12077)
Supplement: Supplemental Information 19 [file peerj-09-12077-s019.docx]

**Raw Data Description / Detail**

**Note:**  All the figures in raw data depict / represent as complete structure except where explained separately.

| **ET (Embryo Transfer fertility) Group Wise fertility** | | | |
| --- | --- | --- | --- |
| **Serial** | **Code** | **Detailed Description** | **Remarks** |
|  | Overall | Breed of Embryo being transferred |  |
|  | Protocol | X1 & FI (CIDR-GnRH in XB and HF cows respectively.  X2 & F2 (CIDR-GnRH in XB and HF Cows) |  |
|  | Recipient ID | Local identification tag / number of recipient cow |  |
|  | Quality | Means Embryo quality. A depicts “A grade” while “M & B depict Morula and Blastocyst” stage of embryo development respectively. |  |
|  | Synchronization | It depicts synchronization between “days after standing heat of recipient” and “days after first AI and embryo collection of donor cows”. |  |
|  | Side | It depicts side of ovary. R means “right side ovary” and L means “left side ovary”. |  |
|  | CL | Grades “1 & 2” belong to the quality of CL in recipient cows. Low number means better quality of CL. |  |
|  | Technician | It depicts the person performing ET into recipient cows.  Iqbal = Muhammad Iqbal, A.Q = Abdul Qayum, KM = Khalid Mahmood |  |
|  | Results | It represents pregnancy in the recipients after ET.  I/C or 100 % means pregnant and Empty or 0 % means not-pregnant. |  |
|  |  |  |  |

XB = Crossbred, HF = Holstein Friesian, CIDR = Control internal drug release.

| **Percentage Data Main Sheet / Superovulation Data** | | | |
| --- | --- | --- | --- |
| **Serial** | **Code** | **Detailed Description** | **Remarks** |
|  | Protocol | It indicates superovulation protocol. 1 means CIDR-GnRH protocol. 2 means CIDR-EB protocol. 3 means control group. |  |
|  | Group Code | 1 = CIDR-GnRH in HF cows = 1 F  2 = CIDR-EB in HF Cows = 2 F  3 = Control in HF cows = 3 F  4 = CIDR-GnRH in XB Cows = 1 X  5 = CIDR-EB in XB cows = 2 X  6 = Control in XB cows = 3 X |  |
|  | TSR | It represents Total structures recovered on embryo flushing (TSR = TEs+DG+UFO) |  |
|  | FSR | Fertilized structures recovered (FSR = TE+DG) |  |
|  | TEs | Transferable embryos as per IETS standards |  |
|  | DG | Degenerated embryos (Fertilized ova with poor cell development or dead cellular mass) |  |
|  | UFO | Unfertilized oocyte |  |
|  | D.A.C / Heat (days) | Number of days taken for return to estrus after embryo collection |  |
|  | Follicle | Number of super-estrus follicles (SEF) |  |
|  | Total CL (Right + Left) | Number of corpus luteum (CL) at the time of embryo collection in super-ovulated cows |  |
|  | Wave (Day) | Tentative day of a new follicular wav emergence |  |
|  | Ovulation percentage | Multiple CLs / SEF * 100 |  |
|  | Fertilization percentage | FSR/TSR*100 |  |
